# Ex-Confederates All Right

**Published:** 1894-02

**Authors:** 


### EX-CONFEDERATES ALL RIGHT.

It will be remembered that in our last it was stated that Congressman Sayers had written Dr. Denton, at Austin, that persons who had served in the Confederate army were not eligible to appointment as medical examiners on pension boards, and asked him to recommend some one who was eligible. It will be remembered also that the Austin District Medical Soeiety, in session in this city in December, denounced the ruling, and passed strong resolutions against it. This coming to the knowledge of the commissioner, he emphatically denied, in a letter to Dr. T. J. Tyner, in this city (Dr. Tyner—an ex-Confederate,—having himself been appointed expert Medical Examiner for the Pension Bureau), that he had made any such ruling, and declared that it was his policy to appoint Confederates when properly endorsed, etc. (We regret that this letter was not received here in time to figure in our last editorial, as it put a different phase on the subject and led to complications; for it was a flat contradiction of Mr. Sayers' statement to Dr. Denton.)

The matter was brought to Mr. Sayers' attention, and the following facts were developed:

Dr. F. E. Daniel, an ex-Confederate Surgeon, applied in April last for appointment on the board, and his application was properly endorsed. Mr. Sayers sent it in to the Department, and asked that the appointment be made. He received a reply from the Pension Office, a note, dated July 10, saying: "Dr. F. E. Daniel, whom you recommend for appointment as examining surgeon on the Board at Austin, Texas, is not eligible, he having participated in the late rebellion,'' and requested Mr. Sayers to make another nomination.

Thus it will be seen that Mr. Sayers made no mistake; there was the letter with the Commissioner's name attached.

When these facts were revealed, and Mr. Sayers' attention was called to the conflict in statements by him and the Commissioner, he called at the Pension Office for an explanation. Judge Lochren, the Commissioner, assured him that he had no recollection of having sent, or ordered sent, the letter of July 10; and declared that such was not the policy of the Department. He furthermore assured Mr. Sayers that he would at once appoint Dr. Daniel, and he did so. Thus ends the matter. Yet it is hard to understand how such reply as Mr. Sayers received, should go out in the name of the Commissioner, without his knowledge, or recollection of having authorized it; and the only plausible explanation is that perhaps the head-clerk acted on his own responsibility, and used the *rubber stamp;* a most unwarraūted piece of assumption of authority, if such be the case.

The prompt action of the Austin District Medical Society alone prevented a great injustice being done not only to Dr. D. but to the ex-Confederates as a class, as doubtless this case would have become a precedent.

